# Supplementary material for: Locomotion control during curb descent: Bilateral ground reaction variables covary consistently during the double support phase regardless of future foot placement constraints
Source: PLoS One. 2022 Oct 5;17(10):e0268090. doi: 10.1371/journal.pone.0268090 (PMC9534401; doi:10.1371/journal.pone.0268090)
Supplement: S1 Appendix — (DOCX) [file pone.0268090.s001.docx]

**APPENDIX**

**Uncontrolled manifold (UCM) analysis of the double support phase of gait**

An overview of the UCM analysis of the ground reaction forces and moments during the double support phase of locomotion is provided below. Further details are available in [14].

The UCM method is a computational engine to test the hypothesis that the across-trial co-variation in a set of input variables (identified by the investigator) stabilizes a smaller set of performance variables (PV; also identified by the investigator) that adequately quantify the performance of a motor task. For the double support phase of curb descent, the input variables are the ground reaction forces (GRFs) and the free moments (FMs) about the vertical axis under the two feet. The PVs are the resultant force (F_Resultant_) and moment (M_Resultant_) acting at the whole-body center of mass (CoM), respectively. These PVs vector quantities. Therefore, we performed six separate UCM analyses to assess whether each component of the PV vectors is stabilized by covariation in the input variables that contribute to the component being analyzed. That is, each PV has its own set of input ground reaction variables (GRVs), listed in Table 1.

**Table 1. The performance variables and the associated input variables. The discriminating value for synergy index is the cut off value to evaluate the presence of a synergy for the each UCM analysis. The subscripts ‘R’ and ‘L’ denote the right and left foot, respectively. ‘F’ is force, ‘M’ is moment, and ‘FM’ is free moment.**

| **Performance variable (PV)** | **Input variables** | **Discriminating value (ΔVz *)** |
| --- | --- | --- |
| Resultant force along AP (F_Resultant-AP_) | F_R-AP_, F_L-AP_ | 0 |
| Resultant force along ML (F_Resultant-ML_) | F_R-ML_, F_L-ML_ | 0 |
| Resultant force along vertical (F_Resultant-V_) | F_R-V_, F_L-V_ | 0 |
| Resultant moment about AP axis (M_Resultant-AP_) | F_R-V_, F_L-V_, F_R-ML_, F_L-ML_ | 0.55 |
| Resultant moment about ML axis (M_Resultant-ML_) | F_R-AP_, F_L-AP_, F_R-V_, F_L-V_ | 0.55 |
| Resultant moment about vertical axis (M_Resultant-V_) | F_R-AP_, F_L-AP_, F_R-ML_, F_L-ML_, FM_R_, FM_L_ | 0.80 |

The input variables for each PV are identified from the mathematical constraint between these quantities. For example, the resultant force at the CoM is simply the sum of the GRFs: ΣF_j_ = F_Rj_ + F_Lj_, where the subscripts ‘R’ and ‘L’ denote the right and left foot, respectively, and j = AP, ML or vertical (V) direction. Similarly, the resultant moment at the CoM is the sum of the moments created by the GRFs and the free moment under each foot. We assumed that the GRFs act at the center of pressure (CoP) under each foot, and therefore, the moment arm for computing the moment of force was the CoP-CoM vector. The moment arm varied with time, but we assumed that it was constant across trials at each time-normalized instant of double support.

The partial derivatives of the constraints with respect to the corresponding input variables were computed to obtain the Jacobian for each constraint. The Jacobians define how small changes in the input variables yield changes in the PVs. The null space of each Jacobian is the UCM for that analysis. Changes in the inputs that align with the UCM do not change the corresponding PV. In contrast, changes in inputs orthogonal (ORT) to the UCM change the PV.

The GRFs were normalized by the participant’s weight. The FMs were normalized by the participant’s weight (W) and height (H) to homogenize the space of the input variables for the analysis of resultant moment about the vertical axis. The Jacobians for the resultant force analyses were simply [1 1], and the UCM and ORT manifolds are readily computed using standard linear algebra tools. However, the Jacobians for the resultant moment analyses are functions of the moment arm components. Therefore, these Jacobians were evaluated at the across-trial average values of the moment arm, and the resulting numerical Jacobians were used to obtain the corresponding UCM and ORT manifolds.

The time series of the normalized GRVs during double support from the 15 trials were time normalized. The deviation in the GRVs at time instant (t*) from the corresponding across-trial means are projected onto the corresponding UCM and ORT manifolds, and the variances in these projections yield V_UCM_ (t*) and V_ORT_ (t*). A synergy index (ΔV) and its z-transformed value (ΔVz) for each analysis was obtained from these two variance components at t*:

$\Delta V\left( t^{*} \right)= \frac{\frac{V_{UCM}\left( t^{*} \right)}{n} - \frac{V_{ORT}\left( t^{*} \right)}{m}}{\frac{V_{UCM}\left( t^{*} \right)+V_{ORT}\left( t^{*} \right)}{n+m}}$,

$\Delta V_{z}\left( t^{*} \right)=0.5\times log\left[ \frac{\left| {\Delta V}_{lower} \right|+\Delta V\left( t^{*} \right)}{\left| {\Delta V}_{upper} \right|-\Delta V\left( t^{*} \right)} \right]$,

where n and m are the dimensions of UCM and ORT manifold, respectively. The synergy index was z transformed since it is bounded: ΔV_lower_ ≤ ΔV ≤ ΔV_upper_. For the resultant force analyses, n = m = 1, ΔV_lower_ = -2, and ΔV_upper_ = 2. Furthermore, ΔV = 0 yields ΔVz = 0, which is the discriminating value indicating the presence of a synergy (ΔVz > 0), an anti-synergy (ΔVz < 0), or a lack of task-specific covariation in the GRFs (ΔVz = 0). For the analyses of resultant moments about the AP and ML axes, n = 3, and m = 1. Therefore, ΔV_lower_ = -4, and ΔV_upper_ = 4/3, and ΔV = 0 yields the discriminating value ΔVz = 0.55. For the analysis of the resultant moment about the vertical axis, n = 5 and m = 1. Therefore, ΔV_lower_ = -6, and ΔV_upper_ = 6/5, and ΔV = 0 yields a discriminating value of ΔVz = 0.80.

This procedure yielded a time series of the ΔVz. We averaged this time series across the double support phase for each task, and used the averages in our statistical analyses.
